# Stem Cell Therapy for Autism

**DOI:** 10.1186/1479-5876-5-30

**Published:** 2007-06-27

**Authors:** Thomas E Ichim, Fabio Solano, Eduardo Glenn, Frank Morales, Leonard Smith, George Zabrecky, Neil H Riordan

**Affiliations:** 1 Medistem Laboratories Inc, Tempe, Arizona, USA; 2 Institute for Cellular Medicine, San Jose, Costa Rica; 3 Americas Medical Center, Ridgefield, Connecticut, USA; 4 2027 E. Cedar Street Suite 102 Tempe, AZ 85281, USA

## Abstract

Autism spectrum disorders (ASD) are a group of neurodevelopmental conditions whose incidence is reaching epidemic proportions, afflicting approximately 1 in 166 children. Autistic disorder, or autism is the most common form of ASD. Although several neurophysiological alterations have been associated with autism, immune abnormalities and neural hypoperfusion appear to be broadly consistent. These appear to be causative since correlation of altered inflammatory responses, and hypoperfusion with symptology is reported. Mesenchymal stem cells (MSC) are in late phases of clinical development for treatment of graft versus host disease and Crohn's Disease, two conditions of immune dysregulation. Cord blood CD34+ cells are known to be potent angiogenic stimulators, having demonstrated positive effects in not only peripheral ischemia, but also in models of cerebral ischemia. Additionally, anecdotal clinical cases have reported responses in autistic children receiving cord blood CD34+ cells. We propose the combined use of MSC and cord blood CD34+cells may be useful in the treatment of autism.

## Background

Autism spectrum disorders (ASD) are reaching epidemic proportions, believed to affect approximately 1 in 166 children. Autism, Asperger's syndrome, Rett's disorder, and childhood disintegrae disorder are all encompassed by the term ASD. Autism is the most prevalent ASD, characterized by abnormalities in social interaction, impaired verbal and nonverbal communication, and repetitive, obsessive behavior. Autism may vary in severity from mild to disabling and is believed to arise from genetic and environmental factors. While symptomology of autism may be noted by caregivers around 12–18 months [1], definitive diagnosis generally occurs around 24–36 months, however in some cases diagnosis may be made into adulthood [2]. Determination of autism is performed using the DSM-IV-TR, or other questionnaires and tests. Children with autism appear withdrawn, self-occupied, and distant. Inflexibility in terms of learning from experiences and modifying patterns to integrate into new environments is characteristic of autism. Depending on degree of severity, some children with autism may develop into independent adults with full time employment and self sufficiency; however this is seldom the case.

Current treatments for autism can divided into behavioral, nutritional and medical approaches, although no clear golden standard approach exists. Behavioral interventions usually include activities designed to encourage social interaction, communication, awareness of self, and increase attention. Nutritional interventions aim to restrict allergy-associated dietary components, as well as to supplement minerals or vitamins that may be lacking. Medical interventions usually treat specific activities associated with autism. For example, serotonin reuptake inhibitors (SSRI's) such as fluoxetine, fluvoxamine, sertraline, and clomipramine, are used for treatment of anxiety and depression. Some studies have shown that SSRI's also have the added benefit of increasing social interaction and inhibiting repetitive behavior. Typical antipsychotic drugs such as thioridazine, fluphenazine, chlorpromazine, and haloperidol have been showed to decrease behavioral abnormalities in autism. Atypical antipsychotics such as risperidone, olanzapine and ziprasidone have also demonstrated beneficial effect at ameliorating behavioral problems. Autism associated seizures are mainly treated by administration of anticonvulsants such as carbamazepine, lamotrigine, topiramate, and valproic acid. Attention deficient/hyperactivity is treated by agents such as methylphenidate (Ritalin^®^).

Currently, numerous clinical trials are being conducted with interventions ranging from hyperbaric oxygen, to administration of zinc, to drugs exhibiting anti-inflammatory properties. Unfortunately, no clear understanding of autism's pathogenic mechanisms exists, and as a result numerous strategies are being attempted with varying degrees of success. In this paper we examine two pathologies associated with autism – hypoperfusion to the brain and immune dysregulation – and propose a novel treatment: the administration of CD34+ umbilical cord cells and mesenchymal cells.

## Hypoperfusion of brain in autism

Children with autism have been consistently shown to have impaired, or subnormal CNS circulation, as well as resulting hypoxia. Defects include basal hypoperfusion [3], and decreased perfusion in response to stimuli that under normal circumstances upregulates perfusion [4]. In numerous studies the areas affected by hypoperfusion seem to correlate with regions of the brain that are responsible for functionalities that are abnormal in autism. For example, specific temporal lobe areas associated with face recognition [5], social interaction [6], and language comprehension [7], have been demonstrated to be hypoperfused in autistic but not control children.

The question of cause versus effect is important. If temporal lobe ischemia is not causative but only a symptom of an underlying process, then targeting this pathology may be non-productive from the therapeutic perspective. However this appears not to be the case. It is evident that the degree of hypoperfusion and resulting hypoxia correlates with the severity of autism symptoms. For example, statistically significant inverse correlation has been demonstrated between extent of hypoxia and IQ [8]. Supporting a causative effect of hypoperfusion to autism development, Bachavelier et al reviewed numerous experimental reports of primate and other animal studies in which damage causing hypoperfusion of temporal areas was associated with onset of autism-like disorders [9]. It is also known that after removal or damage of the amygdala, hippocampus, or other temporal structures induces either permanent or transient autistic-like characteristics such as unexpressive faces, little eye contact, and motor stereotypies occurs. Clinically, temporal lobe damage by viral and other means has been implicated in development of autism both in adults [10], and children [11-14].

Evidence suggests that hypoperfusion and resulting hypoxia is intimately associated with autism, however the next important question is whether reversion of this hypoxia can positively influence autism. In autism the associated hypoxia is not predominantly apoptotic or necrotic to temporal neurons but associated with altered function [15]. Hypoperfusion may contribute to defects not only by induction of hypoxia but also allowing for abnormal metabolite or neurotransmitter accumulation. This is one of the reasons why glutamate toxicity has been implicated in autism [16] and a clinical trial at reversing this using the inhibitor of glutamate toxicity, Riluzole, is currently in progress [17]. Conceptually the augmentation of perfusion through stimulation of angiogenesis should allow for metabolite clearance and restoration of functionality. Although not well defined, cell death may also be occurring in various CNS components of autistic children. If this were the case, it is possible that neural regeneration can be stimulated through entry of neuronal progenitor cells into cell cycle and subsequent differentiation. Ample evidence of neural regeneration exists in areas ranging from stroke [18], to subarachinoidal hemorrhage [19,20], to neural damage as a result of congenital errors of metabolism [21]. Theoretically, it is conceivable that reversing hypoxia may lead to activation of self-repair mechanisms. Such neural proliferation is seen after reperfusion in numerous animal models of cerebral ischemia [22-24]. The concept of increasing oxygen to the autistic brain through various means such as hyperbaric medicine is currently being tested in 2 independent clinical trials in the US [25,26]. However, to our knowledge, the use of cell therapy to stimulate angiogenesis has not been widely-used for the treatment of autism.

## Immune deregulation in autism

The fundamental interplay between the nervous system and the immune system cannot be understated. Philosophically, the characteristics of self/nonself recognition, specificity, and memory are only shared by the immune system and the nervous system. Physically, every immune organ is innervated and bi-directional communication between neural and immune system cells has been established in numerous physiological systems. In autism, several immunological abnormalities have been detected both in the peripheral and the central nervous systems.

Astroglial cells, or astrocytes, surround various portions of the cerebral endothelium and play a critical role in regulating perfusion [27,28], and blood brain barrier function [29]. Astrocytes are capable of mediating several immunological/inflammatory effects. Expression of various toll like receptors (TLR) on astrocytes endows the ability to recognize not only bacterial and viral signals but also endogenous "danger" signals such as heat shock proteins, fibrinogen degradation products, and free DNA [30]. Physiologically, astrocytes play an important protective role against infection, generating inflammatory cytokines such as TNF-alpha, IL-1beta, and IL-6 [31]. Through secretion of various chemokines such as CXCL10, CCL2 and BAFF, astrocytes play an important role in shaping adaptive immune responses in the CNS [32]. Astrocytes have antigen presenting capabilities and have been demonstrated to activate T and B cell responses against exogenous and endogenous antigens [33,34]. Although astrocytes play a critical role against CNS infection, these cells also have potential to cause damage to the host when functioning in an aberrant manner. For example, various neurological diseases are associated with astrocyte overproduction of inflammatory agents, which causes neural malfunction or death. In amyotrophic lateral sclerosis (ALS), astrocyte secretion of a soluble neurotoxic substance has been demonstrated to be involved in disease progression [35,36]. Astrocyte hyperactivation has been demonstrated in this disease by imaging, as well as autopsy studies [37-39]. In multiple sclerosis, astrocytes play a key role in maintaining autoreactive responses and pathological plaque formation [40,41]. In stroke, activated astrocytes contribute to opening of the blood brain barrier [42], as well as secrete various neurotoxic substances that contribute to post infarct neural damage [43,44].

Vargas et al compared brain autopsy samples from 11 autistic children with 7 age-matched controls. They demonstrated an active neuroinflammatory process in the cerebral cortex, white matter, and notably in cerebellum of autistic patients both by immunohistochemistry and morphology. Importantly, astrocyte production of inflammatory cytokines was observed, including production of cytokines known to affect various neuronal functions such as TNF-alpha and MCP-1. CSF samples from living autism patients but not controls also displayed upregulated inflammatory cytokines as demonstrated by ELISA [45]. The potent effects of inflammatory cytokines on neurological function cannot be underestimated. For example, patients receiving systemic IFN-gamma therapy for cancer, even though theoretically the protein should not cross the blood brain barrier, report numerous cognitive and neurological abnormalities [46,47]. In fact, IFN-gamma, one of the products of activated astrocytes [46], has been detected at elevated levels in the plasma of children with autism [48,49]. Mechanistically, inflammatory mediators mediate alteration of neurological function through a wide variety of different pathways, either directly altering neuron activity or indirectly. For example, the common neurotoxin used in models of Parkinson's Disease, MPTP is believed to mediate its activity through activation of IFN-gamma production, leading to direct killing of dopaminergic neurons in the substantia nigra. This is evidenced by reduced MPTP neuronal toxicity in IFN-gamma knockout mice or by addition of blocking antibodies to IFN-gamma [50]. In terms of indirect effects of IFN-gamma, it is known that this cytokine activates the enzyme 2,3-indolaminedeoxygenase, leading to generation of small molecule neurotoxins such as the kynurenine metabolites 3OH-kynurenine and quinolinic acid which have been implicated in dementias associated with chronic inflammatory states [51,52].

T cell and B cell abnormalities have been reported systemically in autistic children. These have included systemic T cell lymphopenia, weak proliferative responses to mitogens, and deranged cytokine production [53,54]. At face value, lymphopenia would suggest general immune deficiency and as a result little inflammation, however, recent studies have demonstrated that almost all autoimmune diseases are associated with a state of generalized lymphopenia (reviewed by Marleau and Sarvetnick [55]). Autoimmune-like pathophysiology appears to be prevalent in autism and several lines of reasoning suggest it may be causative. Firstly, numerous types of autoantibodies have been detected in children with autism but not in healthy or mentally challenged controls. These include antibodies to myelin basic protein [56], brain extracts [57,58], Purkinje cells and gliadin extracted peptides [59], neutrophic factors [60,61], and neuron-axon filament and glial fibrillary acidic protein [61]. Secondly, family members of autistic children have a higher predisposition towards autoimmunity compared to control populations [62,63]. Hinting at genetic mechanisms are observations that specific HLA haplotypes seem to associate with autism [64,65]. Another genetic characteristic associated with autism is a null allele for the complement component C4b [66]. Both HLA haplotypes as well as complement component gene polymorphisms have been strongly associated with autoimmunity [67-69]. It is known that autoimmune animals have altered cognitive ability and several neurological abnormalities [70]. Thirdly, autism has been associated with a peculiar autoimmune-like syndrome that is still relatively undefined. Mucosal lesions in the form of chronic ileocolonic lymphoid nodular hyperplasia characterized by lymphocyte infiltration, complement deposition, and cytokine production have been described uniquely to children with autism but not healthy controls or cerebral palsy patients [71]. This inflammatory condition is associated not only with lesions on the intestinal wall, but also in the upper GI tract. Although several characteristics of this condition are shared with Crohn's Disease, one unique aspect is eosinophilic infiltrate, which seems to be associated with dietary habits of the patient [72]. Systemic manifestation of the immune deregulation/chronic inflammatory condition are observed through elevated levels of inflammatory cytokines such as IFN-gamma [73], IL-12 [74], and TNF-alpha [75]. Indication that a relevant inflammatory response is ongoing is provided by observation that the macrophage product neopterin is observed elevated in children with autism [76]. Inhibited production of anti-inflammatory cytokines such as IL-10 [77] and TGF-beta [78] has also been observed in children with autism, thus suggesting not only augmentation of inflammatory processes but also deficiency of natural feedback inhibitor mechanisms.

The systemic effects of a chronic inflammatory process in the periphery may result in production of soluble factors such as quinilonic acid, which have neurotoxin activity. Ability of cellular immune deregulation to affect neural function can occur independent of cell trafficking, as was demonstrated in animal studies in which T cell depletion was associated with cognitive loss of function that was reversible through T cell repletion [79]. Localized inflammation and pathological astrocyte activation has been directly demonstrated to be associated with pathogenesis in autism. Clinical trials of inflammatory drugs have demonstrated varying degrees of success. For example, in an open labeled study of the anti-inflammatory PPAR-gamma agonist pioglitozone in 25 children, 75% reported responses on the aberrant behavior checklist [80]. Other interventions aimed at reducing inflammation such as intravenous immunoglobulin administration reported inconsistent results, however a minor subset did respond significantly [81,82]. Clinical trials are currently using drugs off-label for treatment of autism through inhibiting inflammation such as minocycline [83], n-acetylcysteine [84], or ascorbic acid and zinc [85]. Despite the desire to correct immune deregulation/chronic inflammation in autism, to date, no approach has been successful.

## Treatment of hypoperfusion defect by umbilical cord blood CD34+ stem cell administration

Therapeutic angiogenesis, the induction of new blood vessels from preexisting arteries for overcoming ischemia, has been experimentally demonstrated in peripheral artery disease [86], myocardial ischemia [87], and stroke [88]. Angiogenesis is induced through the formation of collateral vessels and has been observed in hypoperfused tissues. This process is believed to be coordinated by the oxygen sensing transcription factor hypoxia inducible factor-1 (HIF-1). During conditions of low oxygen tension, various components of the transcription factor dimerize and coordinately translocate into the nucleus causing upregulation of numerous cytokines and proteins associated with angiogenesis such as SDF-1, VEGF, FGF, and matrix metalloproteases [89]. The potency of tissue ischemia stimulating angiogenesis is seen in patients after myocardial infarction in which bone marrow angiogenic stem cells mobilize into systemic circulating in response to ischemia induced chemotactic factors [90]. The angiogenic response has also been demonstrated to occur after cerebral ischemia in the form of stroke and is believed to be fundamental in neurological recovery [91]. For example, in models of middle cerebral artery occlusion, endogenous angiogenesis occurs which is also involved in triggering migration of neural stem cells into damaged area that participate in neuroregeneration [92]. The association between neural angiogenesis and neurogenesis after brain damage is not only temporally-linked but also connected by common mediators, for example, SDF-1 secreted in response to hypoxia also induces migration of neural progenitors [92]. Angiogenic factors such as VEGF and angiopoietin have been implicated in post ischemia neurogenesis [93].

While recovery after cerebral ischemia occurs to some extent without intervention, this recovery is can be limited. Methods to enhance angiogenesis and as a result neurogenesis are numerous and have utilized approaches that upregulate endogenous production of reparative factors, as well as administration of exogenous agents. For example, administration of exogenous cytokines such as FGF-2 [94], erythropoietin [95], and G-CSF [96], has been performed clinically to accelerate healing with varying degrees of success.

A promising method of increasing angiogenesis in situations of ischemia is administration of cells with potential to produce angiogenic factors and the capacity to differentiate into endothelial cells themselves. Accordingly, the use of CD34+ stem cells has been previously proposed as an alternative to growth factor administration [97]. Therapeutic administration of bone marrow derived CD34+ cells has produced promising results in the treatment of end-stage myocardial ischemia [98], as well as a type of advanced peripheral artery disease called critical limb ischemia [99]. Additionally, autologous peripheral blood CD34+ cells have also been used clinically with induction of therapeutic angiogenesis [100]. Of angiogenesis stimulating cell sources, cord blood seems to possess CD34+ cells with highest activity in terms of proliferation, cytokine production, as well as endothelial differentiation [101,102].

Cord blood has been used successfully for stimulation of angiogenesis in various models of ischemia. In one report, the CD34+, CD11b+ fraction, which is approximately less than half of the CD34+ fraction of cord blood was demonstrated to possess the ability to differentiate into endothelial cells [102]. In another report, VEGF-R3+, CD34+ cells demonstrated the ability to differentiate into endothelial cells and were able to be expanded 40-fold expansion. The concentration of this potential endothelial progenitor fraction in cord blood CD34+ cells is approximately tenfold higher as compared to bone marrow CD34+ cells (1.9% +/- 0.8% compared to 0.2% +/- 0.1%) [103]. Administration of cord blood CD34+ cells into immune compromised mice that underwent middle cerebral artery ligation reduced neurological deficits and induce neuroregeneration, in part through secretion of angiogenic factors [104]. Several studies have confirmed that systemic administration of cord blood cells is sufficient to induce neuroregeneration [105-107]. Given the potency of cord blood CD34+ cells to induce angiogenesis in areas of cerebral hypoperfusion, we propose that this cell type may be particularly useful for the treatment of autism, in which ischemia is milder than stroke induced ischemia, and as a result the level of angiogenesis needed is theoretically lower. However at face value, several considerations have to be dealt with. Firstly, cord blood contains a relatively low number of CD34+ cells for clinical use. Secondly, very few patients have access to autologous cord blood; therefore allogeneic cord blood CD34+ cells are needed if this therapy is to be made available for widespread use. There is a belief that allogeneic cord blood cells can not be used without immune suppression to avoid host versus graft destruction of the cells.

Numerous laboratories are currently attempting to expand cord blood CD34+ cells, achieving varying degrees of success. Expansion methods typically involve administration of cytokines, and or feeder cell layers [108-110]. The authors have developed a CD34+ expansion protocol that yields up to 60-fold expansion with limited cell differentiation. This expansion method involves numerous growth factors and conditioned medium, however is performed under serum free conditions (manuscript in preparation). Currently over 100 patients have been treated by one of the authors (FS) with expanded CD34+ cells under local ethical approval with varying degrees of success. Since other groups are also generating CD34+ expansion technologies, we do not anticipate number of CD34+ cells to be a problem.

Safety concerns regarding allogeneic CD34+ cells are divided into fears of graft versus host reactions, as well as host versus graft. The authors of the current paper have recently published a detailed rationale for why administration of cord blood cells is feasible in absence of immune suppression [111]. Essentially, GVHD occurs in the context of lymphopenia caused by bone marrow ablation. Administration of cord blood has been reported in over 500 patients without a single one suffering GVHD if no immune suppression was used [112-115]. Although host versus graft may conceptually cause immune mediated clearing of cord blood cells, efficacy of cord blood cells in absence of immune suppression has also been reported [116-118]. Accordingly, we believe that systemic administration of expanded cord blood derived CD34+ cells may be a potent tool for generation of neoangiogenesis in the autistic brain.

## Immune modulation by mesenchymal stem cells

The treatment of immune deregulation in autism is expected to not only cause amelioration of intestinal and systemic symptomology, but also to profoundly influence neurological function. Reports exist of temporary neurological improvement by decreasing intestinal inflammation through either antibiotic administration [119] or dietary changes [120]. Although, as previously discussed, some anti-inflammatory treatments have yielded beneficial effects, no clinical agent has been developed that can profoundly suppress inflammation at the level of the fundamental immune abnormality. We believe mesenchymal stem cell administration may be used for this purpose. This cell type, in allogeneic form, is currently in Phase III clinical studies for Crohn's disease and Phase II results have demonstrated profound improvement [121].

Mesenchymal stem cells are classically defined as "formative pluripotential blast cells found inter alia in bone marrow, blood, dermis and periosteum that are capable of differentiating into any of the specific types of mesenchymal or connective tissues. These cells are routinely generated by culture of bone marrow in various culture media and collection of the adherent cell population. This expansion technique is sometimes used in combination with selection procedures for markers described above to generate a pure population of stem cells. An important characteristic of mesenchymal stem cells is their ability to constitutively secrete immune inhibitory factors such as IL-10 and TGF-b while maintaining ability to present antigens to T cells [122,123]. This is believed to further allow inhibition of immunity in an antigen specific manner, as well as to allow the use of such cells in an allogeneic fashion without fear of immune-mediated rejection. Antigen-specific immune suppression is believed to allow these cells to shut off autoimmune processes. Further understanding of the immune inhibitory effects of mesenchymal stem cells comes from the fact that during T cell activation, two general signals are required for the T cell in order to mount a productive immune response, the first signal is recognition of antigen, and the second is recognition of costimulatory or coinhibitory signals. Mesenchymal cells present antigens to T cells but provide a coinhibitory signal instead of a co-stimulatory signal, thus specifically inhibiting T cells that recognize them, and other cells expressing similar antigens. Supporting this concept, it was demonstrated in a murine model that mesenchymal stem cell transplantation leads to permanent donor-specific immunotolerance in allogeneic hosts and results in long-term allogeneic skin graft acceptance [124]. Other studies have shown that mesenchymal stem cells are inherently immunosuppressive through production of PGE-2, interleukin-10 and expression of the tryptophan catabolizing enzyme indoleamine 2,3,-dioxygenase as well as Galectin-1 [125,126].

These stem cells also have the ability to non-specifically modulate the immune response through the suppression of dendritic cell maturation and antigen presenting abilities [127,128]. Immune suppressive activity is not dependent on prolonged culture of mesenchymal stem cells since functional induction of allogeneic T cell apoptosis was also demonstrated using freshly isolated, irradiated, mesenchymal stem cells [129]. Others have also demonstrated that mesenchymal stem cells have the ability to preferentially induce expansion of antigen specific T regulatory cells with the CD4+ CD25+ phenotype [130]. Supporting the potential clinical utility of such cells, it was previously demonstrated that administration of mesenchymal stem cells inhibits antigen specific T cell responses in the murine model of multiple sclerosis, experimental autoimmune encephalomyelitis, leading to prevention and/or regression of pathology [131]. Safety of infusing mesenchymal stem cells was illustrated in studies administering 1–2.2 × 10^6 ^cells/kg in order to enhance engraftment of autologous bone marrow cell. No adverse events were associated with infusion, although level of engraftment remained to be analyzed in randomized trials [132]. The ability of mesenchymal stem cells on one hand to suppress pathological immune responses but on the other hand to stimulate hematopoiesis leads to the possibility that these cells may also be useful for treatment of the defect in T cell numbers associated with autism.

## Practical clinical entry

We propose a Phase I/II open labeled study investigating combination of cord blood expanded CD34+ cells together with mesenchymal stem cells for the treatment of autism. Such a trial would utilize several classical instruments of autism assessment such as the Aberrant Behavior Checklist and the Vineland Adaptive Behavior Scale (VABS) for assessment of symptomatic effect. Objective measurements of temporal lobe hypoperfusion, intestinal lymphoid hypertrophy, immunological markers and markers of hypoxia will be included. In order to initiate such an investigation, specific inclusion/exclusion criteria will be developed taking into account a population most likely to benefit from such an intervention. Criteria of particular interest would include defined hypoxia areas, as well as frank clinical manifestations of inflammatory intestinal disease. Markers of inflammatory processes may be used as part of the inclusion criteria, for example, elevation of C-reactive protein, or serum levels of TNF-alpha, IL-1, or IL-6 in order to specifically identify patients in whom the anti-inflammatory aspects of stem cell therapy would benefit [133,134]. More stringent criteria would include restricting the study to only patients in which T cell abnormalities are present such as ex vivo hypersecretion of interferon gamma upon anti-CD3/CD28 stimulation [135], as well as deficient production of immune inhibitory cytokines such as IL-10 [77] and TGF-beta [78].

One of the authors (FS) has utilized both CD34+ and mesenchymal stem cells clinically for treatment of various diseases. In some case reports, the combination of CD34+ and mesenchymal stem cells was noted to induce synergistic effects in neurological diseases, although the number of patients are far too low to draw any conclusions. We propose to conduct this study based on the previous experiences of our group in this field, as well as numerous other groups that have generated anecdotal evidence of stem cell therapy for autism but have not published in conventional journals. We believe that through development of a potent clinical study with appropriate endpoints, much will be learned about the pathophysiology of autism regardless of trial outcome.
